# Preferences for Advanced Therapy Medicinal Products: Understanding the Published Literature on the Value of Innovative Health Interventions

**DOI:** 10.1177/00469580251390763

**Published:** 2025-11-01

**Authors:** N. Ferizović, P. Lorgelly, C. S. Clarke, R. M. Hunter, R. Plackett, W. Abbas, N. Freemantle

**Affiliations:** 1 University College London, UK; 2 University of Auckland, New Zealand

**Keywords:** advanced therapy medicinal product, attributes, reimbursement, preferences, value

## Abstract

Advanced therapy medicinal products (ATMPs) are ground-breaking genetic and cell/tissue-based therapies with the potential to treat and even cure myriad health conditions. However, they have faced multiple challenges in decision making with relatively low success in achieving reimbursement. The aim of this study was to review the published preference-based research on ATMPs to identify value attributes as a starting point in a research workstream aimed at understanding the true value of these innovative therapies. A literature review was used to identify studies across MedLine, Embase, Allied and Complementary Medicine Database, Global Health, ProQuest Central, Social Sciences Premium Collection, Applied Social Sciences Index and Abstracts, Econ Lit, Web of Science and SCOPUS from inception to 3rd March 2024. Search terms for genetic and cell/tissue-based therapies were conducted separately. Studies were included if they were a peer-reviewed primary research study or systematic literature review using stated preference measures to elicit preferences for ATMPs. Thirteen studies were included with 8 pertaining to genetic therapies and 5 to cell/tissue therapies. Attributes of value were similar across the studies and ATMP categories. Attributes were grouped into the following for genetic therapies: clinical benefits, uncertainty, risk, treatment burden, and quality of life; for cell/tissue therapies clinical benefit, uncertainty, risk and treatment burden. There is limited research on the preferences for ATMPs; but these findings suggest that the value attributes of ATMPs are consistent across gene and cell/tissue therapies with clinical benefit, uncertainty, risk, treatment burden, and quality of life being important.

**Highlights**

● This literature review aimed to identify the published literature on preference studies of ATMPs to understand the value attributes of ATMPs and how they are quantified, and to contribute to research to determine whether current HTA methods capture the relevant attributes of value.

● This review has found that ‘clinical benefit’, ‘uncertainty/risk’, and ‘treatment burden’, and ‘quality of life’ are important value attributes to stakeholders – although this conclusion must account for the limited published evidence in this space in that the findings will require validation.

● Although the studies identified suggest that value attributes between the categories of ATMP are broadly similar, in light of the relatively low number of studies identified, further research is needed to explore this and to confirm any similarities and dissimilarities to ensure fair assessment of these therapies and to understand whether current HTA methods suffice.

● Additional research aimed at understanding the broader value of ATMPs and the attributes of value associated with these innovative therapies is necessary. This should include additional stakeholders beyond those of clinicians and patients to ensure all relevant value attributes are considered in HTA to ensure appropriate value assessment of ATMPs.

## Introduction

Advanced therapy medicinal products (ATMPs) are ground-breaking treatment options which include gene therapies, somatic cell therapies or tissue engineered products with the potential to cure a myriad of health conditions.^1,2^ These range from rare diseases with limited treatment options to chronic conditions such as in oncology.^3^ To date, 21 ATMPs have been approved by the European Medicines Agency (EMA)^4^ while the United States (US) Food and Drug Administration (FDA) has approved 46.^5^ However, despite their curative potential, their adoption into mainstream healthcare has been limited with pricing and reimbursement challenges being key factors. A reason for this is that there is uncertainty surrounding the value that these innovative medicines bring to patients and society and whether they warrant the relatively high prices.^6-10^

Many countries undertake an assessment of healthcare interventions, including ATMPs, through a process known as health technology assessment (HTA) which is used to inform decision making in healthcare.^11^ This interdisciplinary process assesses the clinical, economic, legal and societal impacts of an intervention as well as its price.^12^ In many countries, the outcome of this assessment determines patient access to health interventions with ATMPs having a relatively low success rate in achieving full reimbursement.^13^

One of the reasons for this may be that current HTA methods are not suited for value assessment of ATMPs.^14^ As such, there may be a disconnect between existing value assessment frameworks which HTA agencies employ and the potential value ATMPs bring to patients and society.

Thus, understanding the attributes of value of ATMPs from a multi-stakeholder perspective may provide information which would help economists to understand whether current HTA processes capture the full value of ATMPs.

This literature review aimed to identify the published literature on preference studies of ATMPs to understand the value attributes of ATMPs and how they are quantified, and to contribute to research to determine whether current HTA methods capture the relevant attributes of value. This study is part of a larger research workstream with its findings intended to contribute to understanding the value these innovative therapies may bring to patients and wider society. The findings will help to inform whether a review of HTA methodologies specific to ATMPs is needed.

## Methods

This literature review was conducted and reported in accordance with the Preferred Reporting Items for Systematic Reviews and Meta-Analyses (PRISMA) statement to ensure study robustness.^15^ The definition of advanced therapy medicinal products applied was that of the European Medicines Agency (EMA).^16^ In summary, ATMPs are classed into 3 categories: ‘gene therapy medicines which contain genes that lead to a therapeutic, prophylactic or diagnostic effect’, ‘somatic cell therapy medicines contain cells or tissues that have been manipulated to change their biological characteristics or cells or tissues not intended to be used for the same essential functions in the body’, and ‘tissue-engineered medicines which contain cells or tissues that have been modified so they can be used to repair, regenerate or replace human tissue’. Combined ATMPs consisting of 1 of the above 3 categories in combination with a medical device is the final category of ATMP recognised by the EMA.^16^

### Review Aims

The aims of this literature review were twofold:

- To identify the available research on quantitative stated preference studies for ATMPs.
- To identify the available research on qualitative development studies which informed quantitative preference studies (ie, these were studies which informed the development of stated preference studies but contained no quantitative assessment themselves).

This review included quantitative preference studies as this enables an understanding of the quantification of value attributes while qualitative studies provide evidence on potential attributes of value.

The review was not limited to any specific stakeholder, disease area or geography.

The review intended to narratively synthesise the findings of the identified studies with findings separated in line with the type of study – quantitative or qualitative – regardless of methodology used to study preferences or qualitative methodology applied, respectively. This was done in order to amalgamate the findings from the studies.

### Search Strategy

The searches were conducted separately for genetic therapies and cell- and tissue-based therapies. The search strings contained a combination of MeSH terms, title/abstract and free text terms and were designed to be comprehensive to ensure that all relevant studies would be identified. For genetic therapies, terms specific to genetic therapies were used: ‘gene therapy’, ‘personalised medicine’, ‘genetic’, and ‘genomic’. For cell- and tissue-based therapies, terms specific to these therapies included: ‘stem cells’, ‘tissue engineering’, and ‘regeneration’. All search strategies contained terms related to preference elicitation such as the methodologies used to elicit preferences (time trade-off, willingness-to-pay etc.) and methods in which they might be incorporated for example, economic evaluation. The full search terms used in each database as given below are provided in Appendix 1.

The search for relevant literature was conducted in 2021 and 2024 as an update to the original search of 2021 was required due to a delay in the research, as described below.

#### Initial Search

MedLine, Embase, Allied and Complementary Medicine Database, ProQuest Central, Social Sciences Premium Collection, Applied Social Sciences Index and Abstracts, Econ Lit, Web of Science, and SCOPUS from inception to 28th May 2021 were searched.

#### Updated Search

The search for MedLine, Embase, Allied and Complementary Medicine Database, ProQuest Central, Social Sciences Premium Collection, Applied Social Sciences Index and Abstracts, Econ Lit, Web of Science, and SCOPUS was re-run to include publications listed in the database from 1st January 2021 to 3rd March 2024. It was noted that there had been a change in the thesauri for Allied and Complementary Medicine Database and Applied Social Sciences Index and Abstracts thus requiring updated search terms.

The searches were also run in Global Health. For Global Health, it was found that when running the search from inception to March 2024, with the updated search terms the same results as previously identified were not present. This led to Global Health only being included in the updated search covering inception to March 2024.

For any records identified in the overlapping period (ie, 1st January 2021 and 28th May 2021), the records were included in the initial search. That is, they were considered duplicates and excluded.

#### Selection Criteria

All studies were screened against the eligibility criteria as given in Table 1.

**Table 1.** PICOS Criteria.

Parameter	Inclusion	Exclusion
Publication type	Peer-reviewed primary research study or systematic literature review using stated preference measures to elicit preferences for advanced therapy medicinal products.	All other publication types.
	Qualitative studies informing quantitative preference studies.	
Population	Human	non-Human
Intervention	Advanced therapy medicinal product in line with the definition of the European Medicines Agency	All other interventions
Comparators	No restriction	None
Outcomes	No restriction	None
Study design	Quantitative (stated) preference elicitation studies of any type (Best-worst scaling, swing weighting, time trade-off, standard gamble, discrete choice experiment or conjoint analysis, contingent valuation, threshold technique, person trade-off, visual analogue scale, and vignette studies) or qualitative study types (ie, where protocols/interviews are being described as a first step in preference elicitation) or economic evaluations where preferences may be described	• Animal studies
		• In vitro/in vivo studies
		• Editorials
		• Letters
		• Comments
		• Notes
		• Errata
Geographical location	No restriction	None
Language	English	Languages other than English
Publication date	No restriction	None

The Population, Intervention, Comparison, Outcomes, and Study design (PICOS) criteria as given in Table 1 were applied to both treatment types to identify relevant studies. In summary, all peer-reviewed primary research study or literature review using stated preference measures to elicit preferences for advanced therapy medicinal products or qualitative studies informing quantitative preference studies were included. Studies were limited to the English language.

#### Screening and Data Extraction

Due to the high volume of records resulting from the initial search, 1 investigator (NFe) initially screened all titles for eligibility. This has been shown to have no impact on the findings of reviews.^17^ All eligible abstracts were then screened by 2 investigators (NFe and WA) with the resulting eligible full texts screened by NFe.

For the updated search, as there were fewer records for screening, NFe screened the titles and abstracts simultaneously followed by all eligible full texts. Texts were screened against the PICOS criteria (Table 1) with articles not meeting these criteria being excluded. All articles meeting the criteria at full-text review were included in a pre-developed extraction grid used to extract relevant information: author, year, study type, geographical location/setting, sampling and sample size, disease area, methodology, analysis methods, attributes, preferences elicited.

#### Hand-searching

Reference lists of identified relevant systematic literature reviews were hand-searched with relevant references pearl-grown, until no new records were identified. Additionally, we searched Google Scholar using terms synonymous with ‘preferences for advanced therapies’ including ‘preferences for gene therapy/cell therapy/tissue therapy’ to identify relevant studies which may not have been indexed in the databased included in the search strategy.

Further, the International Society for Pharmacoeconomics and Outcomes Research conference proceedings were searched for studies not already identified in the database search or the hand-searching.

#### Quality Review of Included Studies

There are limited quality assessment tools available for stated preference studies with the exception of a checklist developed for conjoint analysis studies.^18^ The results of a summary of compliance against this pre-defined checklist in light of limited methods for assessing preference studies is provided in Appendix 2.

## Results

### Search Results – Genetic Therapies

The initial search identified a total of 6010 records; 391 duplicates were removed. Due to the volume of studies identified, an initial title screen was conducted by NFe to improve the efficiency of the review with no loss of robustness.^17^ About 5619 titles were screened leading to the exclusion of 5577 due to lack of relevance. Subsequently, 42 abstracts were screened leading to the exclusion of 15. A total of 27 full texts were then screened. Twenty-five were excluded giving 2 records for data extraction and evidence synthesis.

From the March 2024 updates, 704 records were identified with 43 duplicates removed. This resulted in 661 titles and abstracts being screened. For the updated search, it was not necessary to assess titles only as a first step due to the lower number of identified studies. Six hundred forty-eight were excluded with 13 eligible for full text screening. Thirteen full-text articles were excluded at full-text screening with no records included for data extraction.

Overall, from the initial and updated database-based searches, 2 records were included in the data extraction and evidence synthesis.

A further 6 records were identified from the hand-search. The authors sought to understand why these 6 records were not identified in the database searches. Following investigation, it was found that the 6 studies were not indexed in the databases at the time the searches were run.

**Figure 1.**
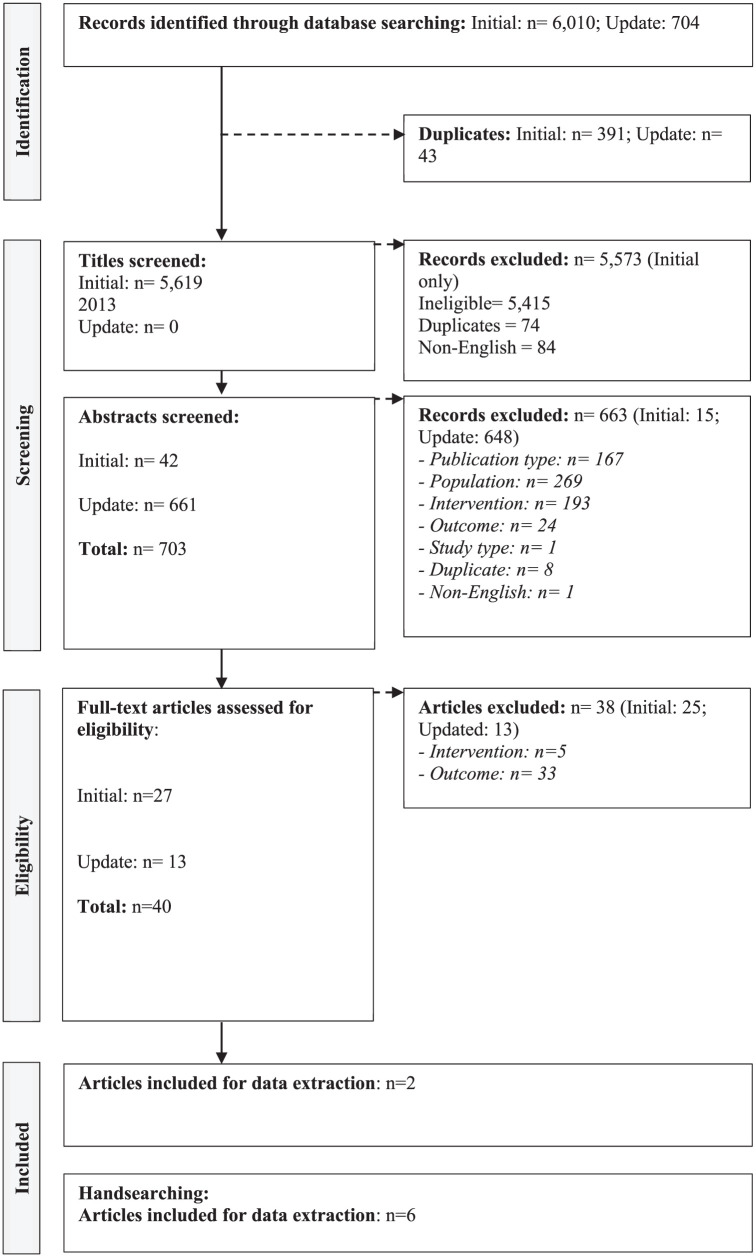
PRISMA: genetic therapies.

The PRISMA for genetic therapies is presented in Figure 1.

### Search Results – Cell and Tissue-Based Therapies

The initial database searches for cell and tissue-based therapies gave a total of 8846 records. Three hundred six of these were duplicates which were removed. Similar to the original search for genetic therapies, the titles were initially screened by NFe.^17^ Following removal of duplicates, 8540 titles were screeded with 63 proceeding into abstract sifting meaning that 8477 were excluded due to not meeting the PICOS inclusion criteria. Of the 63 abstracts screened, 22 were excluded with 41 entering full-text screening. Forty were excluded resulting in 1 record included for data extraction and evidence synthesis.

From the March 2024 database updates, similar to that of genetic therapies, an initial title screen was not conducted as 1838 records were identified. Fifteen of these were duplicates resulting in 1823 records eligible for title and abstract screening. This resulted in 20 full texts being screened as 1803 records were excluded for ineligibility at the title and abstract screening stage. Nineteen full-text articles were excluded. One record was included for data extraction.

Overall, from the database-based searches, 2 records pertaining to cell and tissue-based therapies were identified.

A further 3 studies were identified through hand-searching. Similarly, the authors investigated why more studies were identified from the hand-search compared to the database searches. The 3 studies identified from the hand-search were not indexed in the databases at the time the searches were run.

**Figure 2.**
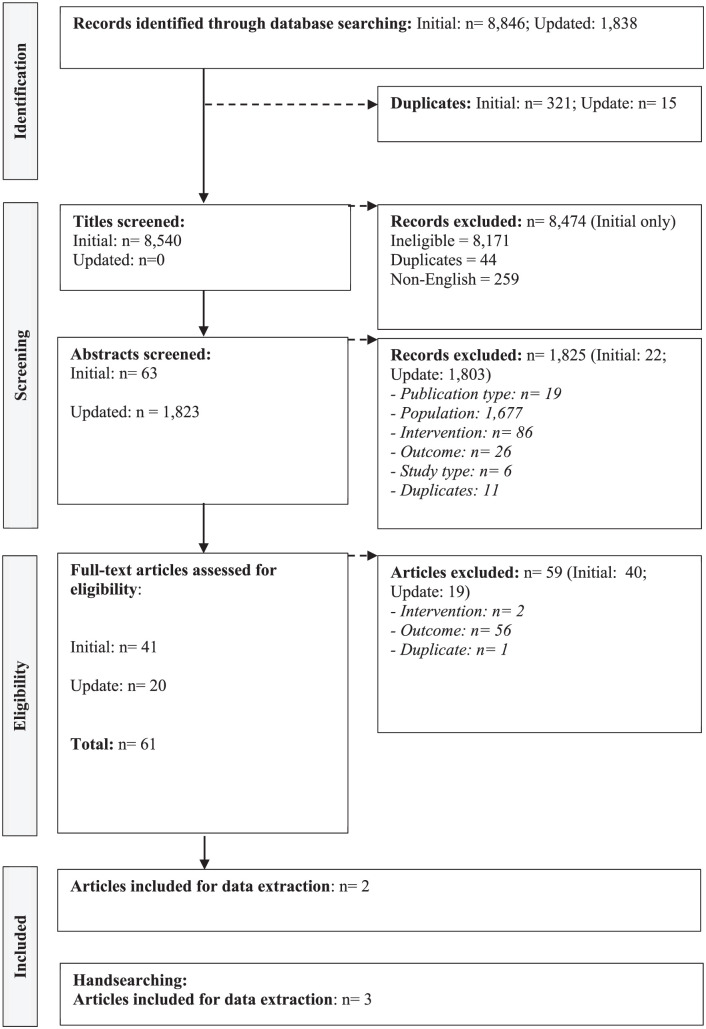
PRISMA: cell and tissue-based therapies.

The PRISMA for cell and tissue-based therapies is presented in Figure 2.

#### Summary of the Evidence

The 2 ATMP category-specific searches and hand-searches identified a total of 13 relevant records. Eight of these related to studies for genetic therapies^19-26^ and 5 to studies for cell and tissue-based therapies.^27-31^ For genetic therapies, 6 of the 8 studies^19-22,25,26^ examined preferences or conducted qualitative research ahead of preference studies for gene therapies in haemophilia with the remaining 2 focussing on sickle cell disease^23^ and Duchenne muscular dystrophy,^24^ respectively. For cell and tissue-based therapies, all identified studies were focussed on oncology with 4 explicitly studying chimeric antigen receptor T-cell (CAR T) therapies in B-cell lymphoma.^28-31^

There was a limited geographical spread with studies being conducted in either Europe or the United States only. Stakeholders considered in the identified studies were limited to clinicians or patients.

The authors would have liked to have conducted a quality assessment of the included studies, but this was not possible due to the lack of assessment tools for stated preference studies. The DCEs were assessed against a conjoint analysis checklist^18^ – this is presented in Appendix 2. Most of the discrete choice experiments were largely compliant but with a lack of description on the methods around how the preferences were elicited.

The results are presented in line with the aims of this literature review. The authors defined categories for value attributes (‘treatment attribute themes’) following data extraction to enable cross-comparison between studies so that findings could be synthesised. The study findings were amalgamated for 2 reasons. Firstly, the low number of studies identified and limited types of preference study conducted precluded the authors from synthesising methodologies separately; secondly, the ultimate aim of the review was to understand the value attributes of importance to stakeholders of ATMPs. This meant that study findings needed to be amalgamated.

#### What is the Available Research on Quantitative Stated Preference Studies for ATMPs?

A summary of the characteristics and findings from the quantitative studies is presented in Table 2.

**Table 2.** Characteristics and Findings of Quantitative Studies.

Included studies	Objectives	Disease area and stakeholder	Data collection and sample size	Method and analysis	Attributes	Preferences	Attribute themes
Genetic therapy							
Witkop et al^21^	To conduct a discrete choice experiment to investigate relative importance and differential preferences for gene therapy in the United States	Haemophilia Stakeholder: Patients Sample size: 183	Online survey between November 2020 and February 2021 Participants identified through patient panels including National Haemophilia Foundation Community Voices in Research and M3 Global Research	Discrete choice experiment Random parameter logit regressions; within attribute difference in preference weights as a percentage of the total difference in preference weights across all attributes	Dosing frequency/durability, effect on annual bleeding, uncertainty related to side effects, impact on daily activities, impact on mental health, and post-treatment requirements	Relative importance ranking: effect on overall annual bleeding rate, dose frequency and durability, uncertainty of short- or long-term safety issues, impact on daily life/physical activity, transformative/mental health impact, post treatment (possibility to undergo minor surgery without need for factor therapy). Ranking and scores are similar between patients with Haemophilia A and Haemophilia B with the exception of dose frequency and durability which is ranked first by Haemophilia B patients with overall annual bleeding rate second. Patient preferred 0 bleeds per year compared to 5; one-time treatment over multiple administrations per week.	Clinical benefits, treatment burden, uncertainty/risk, quality of life^a^
van Overbeeke et al^22^	To understand patient trade-offs for gene therapy versus standard of care in Belgium	Haemophilia Stakeholder: Patients Sample size: 117	Survey Participants identified through email and newsletters between April and May 2020 distributed to three national haemophilia reference centres	Threshold analysis Interval regression Tobit models	‘Annual bleeding rate’, ‘Chance to stop prophylaxis,’ ‘Time that side effects have been studied’, and ‘Quality of life’	Annual bleeding rate (ABR): 88% would prefer gene therapy and 12% would not if ABR were 0 with gene therapy; 61% would prefer gene therapy even if the ABR were the same as standard of care. Chance to stop prophylaxis: 68% would prefer gene therapy and 32% would not if prophylaxis were not needed; 29% would prefer gene therapy even if there were only a 20% or lower chance of stopping prophylaxis. Quality of life: 87% would prefer gene therapy and 13% would not were the quality-of-life score 100 for gene therapy; 61% would prefer gene therapy even if the quality-of-life score were the same as standard of care	Clinical benefits, quality of life^a^
Gonzalez Sepulveda et al^23^	To quantify benefit-risk trade-offs in the United States	Sickle cell disease Stakeholder: Adult patients and caregivers of paediatric patients Sample size: 289	Survey Participants identified through Duke University Health System, the University of North Carolina at Chapel Hill, Lurie Children’s Hospital of Chicago, and Cayenne Wellness a patient advocacy group based in California	Discrete choice experiment Random parameters logit regression models for population level preferences (these were rescaled for comparison); heteroskedastic logit models used to evaluate scale. Preference weight estimates from each random parameter logit model were used to calculate minimum-acceptable benefits	Improved survival, fewer symptoms, reduced risk	Showed preference for improved clinical outcomes (improved survival, greater chance of no symptoms). Preferred lower chance of death but non-discriminatory concerning cancer risk. More severe disease led to preference for gene therapy with milder symptom patients less likely to have gene therapy. Patients with mild symptoms had a higher minimum acceptable benefit for 10%/30% chance of death compared to those with moderate symptoms	Clinical benefits, uncertainty/risk^a^
Sun et al^25^	To assess patient preferences for gene therapy in the United States	Haemophilia Stakeholders: Patients Sample size: 95	Online survey between December 2019 and January 2020 Method of identification not stated	Discrete choice experiment Conditional logit regression model to analyse patient preferences; odds ratios to quantify the impact of specific attribute levels; estimates of relative attribute importance to quantify the influence of each attribute on patient decision making	Efficacy, safety, and administration- related measures (frequency, route, out of pocket expenses)	Administration-related attributes were most important; risk of serious side effects was least important. Relative attribute importance: frequency and route of administration, out of pocket expenses, place of administration, chance of breakthrough bleed within 1 year, FVIII level and dynamics, risk of inhibitor development, risk of serious side effects. Lower frequency of administration and treatment at home over clinic were preferred	Clinical benefits, uncertainty/risk, treatment burden^a^
Cell therapy							
Leuthold et al^27^	To understand patient expectations of stem cell transplant in Switzerland	Oncology Stakeholder: Patients Sample size: 184	Questionnaire Participants where all those who received stem cell transplant before October 2018 identified through the University Hospital of Basel	Time trade-off Determined the minimal survival and cure rates	Survival; potential cure	Patients preferred a survival length of at least 5 or 10 years and a cure rate of 50%; patients would undertake therapy if there were a 1-year survival gain	Clinical benefit^a^
Howell et al^28^	To estimate health state utilities associated with toxicities of CAR T-cell treatments in the United Kingdom	Large B-Cell Lymphoma Stakeholder: clinicians used to develop the vignettes; public valuation Sample size: 218	Not clearly stated: clinicians were interviewed; Edinburgh and London were the setting for the time trade-off Public participants were recruited via newspaper and online advertisements. Following telephone screening, participants who were eligible, interested, and available were scheduled for an in-person interview	Time trade-off Determined societal willingness to trade time in perfect health for health states with varying CAR T related adverse events	Adverse events	Respondents would prefer to trade time in perfect health to avoid CAR T related adverse events (Health State A: 72.0%, Health State B: 75.2%, Health State C: 78.9%, Health State D: 89.0%, Health State E: 78.9% and Health State F: 86.7%). Increased severity of adverse events is correlated with preference to avoid these states and to trade perfect health to avoid them.	Uncertainty/risk^a^
Boeri et al^29^	To evaluate physicians’ willingness to trade off benefits, risks, and time to infusion for CAR T-cell therapy in the United States	Relapsed or refractory diffuse large B-cell lymphoma Stakeholder: Clinicians (oncologists/haematologists) Sample size: 150	Online survey Any clinician-based setting in the United States identified through the Market Research team Dynata	Discrete choice experiments Random parameters logit model used to generate preference weights; used to estimate the maximum acceptable percentage-point increases. Maximum acceptable risk increases calculated as the negative of the ratio between the marginal utility for the attributes and marginal disutility of each risk from the lowest level of that risk considered	Six attributes considered: probability of complete response achieved at 6 months, probability of overall survival at 12 months, probability of overall survival at 24 months, time from leukapheresis to infusion, risk of severe cytokine release syndrome, risk of severe neurological event.	Preference weights were ordered as expected. The greatest change in preference weights was seen when time to infusion was reduced from 113 to 16 days. Physicians were willing to accept increases in risk of adverse event for improved clinical outcomes	Clinical benefits, uncertainty/risk, treatment burden^a^
Birch et al^30^	To quantify patient preferences for second-line therapies including CAR T-cell therapy in the United States and Europe (France, Germany, Italy, Spain, and the United Kingdom)	Diffuse large B-cell lymphoma Stakeholder: Patients Sample size: 224	Online survey Participants were identified through Schlesinger Associates	Discrete choice experiment Random parameters logit model: willingness to accept analysis conducted where logit model was run with 1-year survival as a continuous variable and then calculated the marginal rate of substitution to quantify the amount of improvement in 1-year survival necessary to accept risk of serious adverse events (CRS or neurological event)	Identified from the literature and clinical expert input: 1-year survival, 3-year survival, risk of serious infection, risk of serious cytokine release syndrome, risk of serious neurological event, time until functioning returns to pre-treatment levels	Improved probability of 1 year survival was the most important; followed by reduction in adverse events. In Europe, third most important was decrease in the time to return to pre-treatment level of functioning from 5 to 2 months; in the US, this was change in decreasing survival probability from 1 to 3 years. In the USA, a 13.2 percentage point increase in 1-year survival probability; in Europe, it is 14.5 percentage points	Clinical benefit, uncertainty/risk^a^
Liu et al^31^	To understand how patients value benefits and risks associated with CAR T-cell therapy in the United States	Relapsed or refractory diffuse large B-cell lymphoma Stakeholder: Patients Sample size: 95	Online survey Identification method not explicitly stated	Discrete choice experiment A mixed logit model was used to estimate preference weights, calculate relative attribute importance (RAI), and quantify attribute trade-offs.	Two-year treatment success (probability of being alive and in remission), dosing schedule, administration location, risk of acute treatment reactions (cytokine release syndrome, neurological events), risk of serious infections, and severity of chronic side effects.	Probability of treatment success had the largest influence on treatment preferences (RAI 45.3%), followed by risk of serious infections (RAI 19.6%) and acute treatment reactions (RAI 14.7%). Participants were willing to accept a 69.5% increase in risk of serious infections to increase chance of treatment success from 5% to 45%.	Clinical benefit, uncertainty/risk, treatment burden^a^

Note. ABR = Annual bleeding rate; CAR T = Chimeric antigen receptor therapy.

a Categories defined by author so that studies could be cross-compared.

Regarding the genetic therapy studies, 3 of the studies were discrete choice experiments^21,23,25^ with the final conducting a threshold analysis.^22^ Three of the studies elicited preferences for gene therapy in haemophilia^21,25,26^ and one in sickle cell disease.^23^

In haemophilia, Witkop et al^21^ conducted a discrete choice experiment with adult haemophilia patients in the United States. The analysis found that patients attached the greatest importance to ‘effect on overall bleeding rate’ followed by ‘dose frequency and durability’. Similarly, Sun et al^25^ presented patients with 14 choice cards based on attributes developed through targeted literature reviews and telephone interviews. ‘Administration-related measures’ (‘route and frequency of administration’, ‘out-of-pocket cost’, ‘place of administration’) were the most important class of attribute with ‘risk of serious side effects’ the least important. Overall, these studies indicate that in haemophilia, patients value ‘clinical benefits’, ‘treatment burden’, ‘uncertainty/risk’, and ‘quality of life’.

Finally, in haemophilia, van Overbeeke et al^22^ conducted a threshold analysis in Belgium with 117 patients to determine the point at which patients would prefer a gene therapy over the current standard of care (SoC). These findings are not entirely relevant to this literature review as they do not identify *relative* importance, but they do suggest that patients value ‘clinical benefits’, ‘treatment burden’, ‘quality of life’, and ‘uncertainty/risk’.

In sickle cell disease, Gonzalez Sepulveda et al^23^ aimed to quantify benefit/risk trade-offs in adult patients and parents/caregivers of paediatric patients. The analysis found that patients preferred improved efficacy, including greater improvement in life expectancy and no sickle cell disease symptoms thus suggesting that ‘clinical benefits’, and ‘uncertainty/risk’ are important to patients and their caregivers.

Regarding the cell/tissue-based therapies, all studies examined preferences in oncology with 2 of the studies utilising a time trade-off approach^27,28^ while 3 conducted a discrete choice experiment.^29-31^

Leuthold et al^27^ studied patient preferences using time trade-off among 184 patients at a Switzerland-based hospital who had all received stem cell transplants as part of their oncology treatment. The study authors found that patients preferred a survival length of at least 5 or 10 years and a cure rate of 50% to legitimate haematopoietic stem cell transplant. Interestingly, Howell et al^28^ examined the impact of adverse events of CAR T-cell therapy in large B-cell lymphoma with the valuation using time trade-off from a UK societal perspective. Unsurprisingly, preference was for a health state with no adverse events with the health states with the highest grades of adverse events having the lowest preference. This indicates that with increased severity of adverse events from CAR T-cell therapy, respondents would prefer to trade time in perfect health to avoid living in these burdensome health states. These 2 studies which employed similar methods indicate that ‘clinical benefits’ (including the potential for a cure) and ‘uncertainty/risk’ are key attribute themes.

Similar findings were reported by Boeri et al,^29^ Birch et al,^30^ and Liu et al^31^ who all conducted discrete choice experiments in diffuse large B-cell lymphoma to understand the trade-offs between risks and benefits of CAR T-cell therapies. The latter 2 studies sought the preferences of patients and found that treatment success was the most important attribute.^30,31^ Specifically, patients in both the United States and Europe placed the highest importance on 1-year survival followed by reducing the risk of serious CRS or neurological events. This was followed by 3-year survival for patients in the United States and time to return to the pre-treatment level of functioning in Europe.^30^ This indicates that preferences differ by geography albeit patients in both regions would require at least a 13.2% increase in probability of 1-year survival to accept a 13% chance of serious neurological event or 28% chance of cytokine release syndrome (CRS).^30^ This is supported by Liu et al^31^ who found that patients would accept a 69.5% increase in risk of serious infections to increase chance of treatment success from 5% to 45%. There was no evidence of differences between patients and physicians with Boeri et al^29^ highlighting that physicians also preferred improved outcomes, reduced adverse events and less time to infusion. Additionally, physicians were willing to accept increases in adverse events for improved outcomes.^29^

Overall, these studies highlight that ‘clinical benefit’, ‘uncertainty/risk’, ‘quality of life’, and ‘treatment burden’ are important to stakeholders.

#### What is the Available Research on Qualitative Development Studies Which Informed Quantitative Preference Studies?

Table 3 summarises the characteristics and findings of the qualitative studies. The 4 identified studies were limited to genetic therapies. Three of these were focussed on haemophilia^19,20,26^ and were used to inform discrete choice experiments. It is possible these are linked studies, but different results are presented meaning that the studies are described separately.

**Table 3.** Characteristics and Findings of Qualitative studies.

Included studies	Objectives	Disease	Participant identification	Sample size	Data collection	Methods	Findings	Attribute themes
Morgan et al^19^	To conduct qualitative research to inform a discrete choice experiment in the United Kingdom	Haemophilia	Patient representatives and clinical experts – identification method not stated	24	Semi-structured interviews	Points were assigned to attributes based on the rankings by interviewees	Ranking exercise: Impact on factor level, uncertainty regarding long-term risks, impact on daily life, frequency of monitoring, impact on ability to participate in physical activity	Clinical benefit, quality of life, uncertainty/risk and informed decision making^a^
Woollacott et al^26^	To examine treatment preferences for haemophilia in patients and clinicians in the United Kingdom	Haemophilia	People impacted with haemophilia and clinicians Through The Haemophilia Society	20	Semi-structured interviews (April-June 2020) and focus groups to discuss the top ranked attributes	Thematic framework analysis applied to open question responses and ranking of the 6 most important attributes	Ranking exercise: effect on factor level, uncertainty regarding long-term risks, impact on daily life, frequency of monitoring, impact on ability to participate in physical activity, uncertainty regarding long-term benefits, probability that liver inflammation will develop, place of administration, probability that prophylaxis can be stopped after treatment, dose frequency	Therapeutic option, clinical benefit, uncertainty/risk, treatment burden and quality of life^a^
Munetsi et al^20^	To investigate the factors which influence treatment decision making in haemophilia – no geography stated	Haemophilia	Not stated	9 Publications of 174 journals articles and three poster/abstracts identified	Targeted literature review and semi-structured interviews	Attribute ranking and framework analysis to identify the key attributes/themes	Factor expression after gene therapy, uncertainty/variability in factor expression, time in the mild/normal range, chance of short-term adverse events, immunosuppressive agent	Clinical benefit, uncertainty/risk, and treatment burden^a^
Landrum Peay et al^24b^	To explore factors influencing patients and adult patient preferences for gene therapy in the United States	Duchenne muscular dystrophy	Through Parent Project Muscular Dystrophy Patients with Duchenne muscular dystrophy and parents of paediatric patients with Duchenne muscular dystrophy	23	Vignette^b^ between March and May 2017	Thematic analysis	Participants valued benefits to skeletal muscle, cardiac and pulmonary functioning; over half tolerated 1% risk of death when balanced against progression and limited treatment options. Risk tolerance correlated with disease progression with gene therapy valued more with loss of a function; timing of initiation of gene therapy	Clinical benefit, uncertainty/risk^a^

a Categories defined by author so that studies could be cross-compared.

b This study does not make reference to a subsequent preference-based study but was included as the title suggests findings may be used in a quantitative study.

Morgan et al^19^ and Woollacott et al^26^ employed similar methods and techniques to identify values of attribute for gene therapy in haemophilia including semi-structured interviews with around 20 patients and clinicians each. Ranking exercises were used to determine the relative importance of the attributes. Across the 2 studies the following attribute themes were found to be important: ‘therapeutic option’, ‘clinical benefit’, ‘uncertainty/risk’, ‘treatment burden’, ‘informed decision making’, and ‘quality of life’. These findings are supported by Munetsi et al,^20^ in a likely linked study which described a literature review and interviews to identify value attributes in haemophilia.

Finally, Landrum Peay et al^24^ described qualitative research with parents of paediatric patients and adult patients to identify factors influencing treatment choice in Duchenne muscular dystrophy. A thematic analysis indicated that participants place value on treatments which impact on the skeletal muscle, cardiac and pulmonary functioning with the value increasing with disease progression. Risk tolerance was highest in the later disease stages. Thus, ‘clinical benefits’ and ‘uncertainty/risk’ are important in Duchenne with an indication that patient age impacts value attributes.

#### Comparison of Value Attributes for Gene Therapies and Cell and Tissue-Based Therapies

**Figure 3.**
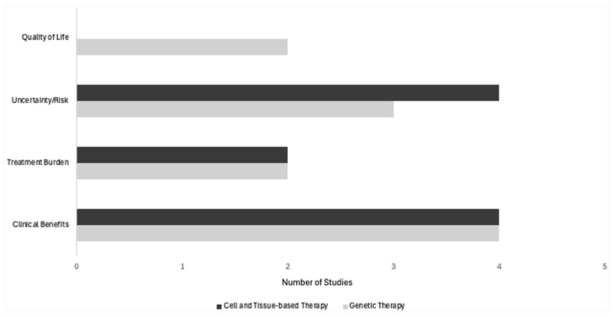
Value attributes by ATMP category.

Figure 3 presents a comparison of the value attribute themes identified in the published quantitative studies. Eight studies in total (4 genetic therapies and 4 cell and tissue-based therapies) identified that clinical benefits are important to patients and clinicians.^21-23,25,27,29-31^ Uncertainty/risk was also important for both categories of ATMP with 3 and 4 studies of genetic and cell/tissue-based therapies, respectively, highlighting that uncertainty/risk play a role in healthcare decision making.^21,23,25,28-31^ Similarly, the burden to patients associated with receiving ATMP treatment was described in an equal number of studies (2 studies each) examining preferences for cell and tissue-based therapies and genetic therapies.^25,29-31^ However, quality of life was only noted in genetic therapy studies (n = 2).^21,22^

## Discussion

This literature review aimed to identify the published literature on preference studies of ATMPs to understand the value attributes of ATMPs and how they are quantified, and to contribute to research to determine whether current HTA methods capture the relevant attributes of value. The review forms part of a larger body of research being conducted to review and inform the development of HTA processes for ATMPs.

This literature review identified 13 quantitative and qualitative studies in total with 8 focussing on genetic therapies^19-26^ and 5 on cell and tissue-based therapies.^27-31^ The quantitative genetic therapy studies were focussed on haemophilia (3 studies; 75%)^21,22,25^ and sickle cell disease (1 study; 25%)^23^ and were limited to discrete choice experiments^21,23,25^ and a threshold analysis.^22^ These studies identified that ‘clinical benefits’, ‘treatment burden’, ‘uncertainty/risk’, ‘quality of life’ were important considerations for stakeholders. The quantitative cell and tissue-based therapy studies were all focussed on oncology.^28-31^ Discrete choice experiments were the preference measure of choice in 3 of the studies^29-31^ with 2 utilising a time trade-off approach.^27,28^ These studies identified that ‘clinical benefit’ (including the potential for a cure), ‘uncertainty/risk’, and ‘treatment burden’ are the most important considerations for stakeholders.^27,29-31^ A comparison between the 2 categories of ATMP found that attributes are widely similar between the categories of ATMP but there is an indication that there are differences between them, namely in the importance of quality of life; however, this may be as a result of the small number of studies identified and with the cell and tissue-based therapy studies focussing on understanding the trade-offs between clinical benefits and adverse events.^28-31^

In terms of the qualitative studies identified, stakeholders expressed that ‘clinical benefit’, ‘quality of life’, ‘uncertainty/risk’ and ‘informed decision making’, ‘treatment burden’, and ‘therapeutic option’ are important considerations for stakeholders. These findings are largely consistent with the findings from the quantitative studies although they carry less weight in the findings of this review. ‘Informed decision making’ and ‘therapeutic option’ were not explored in the quantitative studies identified; this could be because these were not ranked as important attributes ahead of preference elicitation studies.

In summary, ‘clinical benefit’, ‘uncertainty/risk’, and ‘treatment burden’, and ‘quality of life’ were found to be important value attributes to stakeholders with suggestions that other elements such as having an additional treatment option and being informed as part of treatment decisions are also important to stakeholders.^19-31^

These findings must be taken in the context of the studies identified which were of limited geographical spread, restricted to a small number of disease areas and considered a limited number of stakeholders. Specifically, only clinicians and patients were included in the determination of value attributes with studies limited to the United States and Europe.^19-31^ This suggests that study authors considered these stakeholders to be the most important when considering the value of advanced therapies. Conversely, it means that other perspectives have not been considered meaning that the findings of this review are not as comprehensive as intended meaning that applicability is limited and further research is needed. The geographic bias of the identified studies also limits the generalisability of the findings to specific geographies. Finally, the focus on haemophilia, and oncology, limits the representative of the studies and their findings and suggests that further research is needed to decipher whether value attributes of advanced therapies differ by disease area.

This review suggests that current HTA methods capture attributes of value of ATMPs to stakeholders^32-34^ but may not capture all relevant attributes including the potential for a cure and the value of having an alternative treatment option. There may also be additional value attributes which have not been captured in this review particularly in light of the stakeholders considered in the quantification of value being limited to clinicians and patients albeit 1 study utilised a societal perspective but the specific value attributes being valued were defined by clinicians.^28^ It may be that the inclusion of additional stakeholder perspectives such as that of developers, payers, regulators, and the general public would bring to light yet more potentially relevant attributes of value of advanced therapies which are currently not being captured in HTA methods. Indeed, there is evidence that there are other value attributes^14,35,36^ but they have not been included in quantitative ATMP preference elicitation studies; a gap in the field that requires further research.

Although the studies identified as part of this review suggest that value attributes between the categories of ATMP are broadly similar, in light of the relatively low number of studies identified, further research is needed to explore this and to confirm any similarities and dissimilarities to ensure fair assessment of these therapies and to understand whether current HTA methods suffice. Decision makers should be instrumental in discussions around whether current HTA processes are sufficient to ensure that research findings are captured in their value assessment frameworks.

A key strength of this literature review is the comprehensive search strategy both in terms of the search terms and the breadth of databases searched. This ensured a thorough examination of the literature enabling all relevant studies to be identified. This thorough examination identified the lack of research in the understanding of the preferences for ATMPs. To this end, a further key strength of this study is the focus on quantitative preference studies and qualitative studies which contributed to preference studies as this enables all relevant research pertaining to the quantification of value in relation to ATMPs to be identified. Further, the consideration of non-preference-based studies would have meant a different, albeit interesting research question, something that is planned as part of subsequent research.

A drawback of the study is that the title screen for the initial searches was conducted by 1 researcher which may have led to the exclusion of relevant studies. However, the hand-searching will have limited the impact of this. An additional drawback is the lack of robust quality assessment of the included studies which is due to the lack of tools to conduct this assessment. The development of quality checklists for preference studies would help the field to develop robust methods and techniques. A further drawback of this study is that although a thorough assessment of published attributes of value was undertaken, linking these to cost to assess value for money was beyond the study’s scope.

Finally, the low number of studies identified (13 in total) makes it difficult to make firm conclusions from the findings meaning that the strongest conclusion of this study is the need for extensive further research. Further research building on the findings from this study would support the development of a value assessment framework for all ATMPs which could be used in economic evaluation of these health interventions. Indeed, this study forms part of a wider research workstream aiming to develop a value assessment framework for advanced therapies.

## Conclusion

In conclusion, this review has identified the limited published evidence on preference studies in advanced therapies. It has found that ‘clinical benefit’, ‘uncertainty/risk’, and ‘treatment burden’, and ‘quality of life’ are important value attributes to stakeholders – although this conclusion must account for the limited published evidence in this space in that the findings will require validation. However, the review has also identified that there are key gaps in this research space. To ensure that methods used in HTA are relevant for ATMPs and allow their value to be demonstrated, additional research aimed at understanding their broader value and the attributes of value associated with these innovative therapies is necessary. This should include additional stakeholders beyond those of clinicians and patients to ensure all relevant value attributes are considered in HTA to ensure appropriate value assessment of ATMPs. Although there has been some work to date to expand further the attributes of value considered when health interventions are assessed, these are neither mainstream nor do they relate specifically to ATMPs.^36^ There are pre-existing frameworks specifically aimed at advanced therapies although these focus on valuing potential cures and transformative treatments.^35^ HTA agencies should ensure their processes are fair and appropriately assess these innovative therapies.

There is a lack of quality assessment tools for preference studies with this limited to conjoint analysis,^18^ suggesting the strong need for the development of tools to help advance this space. It may be that preference elicitation methods may need to be developed which can be tailored to the complexity of advanced therapies although this will need to be confirmed. This will help to expand the research to emerging markets and diverse healthcare systems to help understand whether value attributes of advanced therapies, and their quantification, are subject to the setting as well as different disease areas. As there is expected to be a large number of advanced therapies entering the market in the next few years, addressing these gaps will need to be swift.^37^

To summarise, there is limited research on the preferences for ATMPs; but these findings suggest that the value attributes of ATMPs are consistent across gene and cell/tissue therapies with clinical benefit, uncertainty, risk, treatment burden, and quality of life being important. However, additional research is needed to confirm and expand on these findings especially in light of the limited number of studies identified. Finally, in order to support the field to advance, new tools and methods will need to be developed.

## Supplemental Material

sj-docx-1-inq-10.1177_00469580251390763 – Supplemental material for Preferences for Advanced Therapy Medicinal Products: Understanding the Published Literature on the Value of Innovative Health Interventions Supplemental material, sj-docx-1-inq-10.1177_00469580251390763 for Preferences for Advanced Therapy Medicinal Products: Understanding the Published Literature on the Value of Innovative Health Interventions by N. Ferizović, P. Lorgelly, C. S. Clarke, R. M. Hunter, R. Plackett, W. Abbas and N. Freemantle in INQUIRY: The Journal of Health Care Organization, Provision, and Financing

sj-docx-2-inq-10.1177_00469580251390763 – Supplemental material for Preferences for Advanced Therapy Medicinal Products: Understanding the Published Literature on the Value of Innovative Health Interventions Supplemental material, sj-docx-2-inq-10.1177_00469580251390763 for Preferences for Advanced Therapy Medicinal Products: Understanding the Published Literature on the Value of Innovative Health Interventions by N. Ferizović, P. Lorgelly, C. S. Clarke, R. M. Hunter, R. Plackett, W. Abbas and N. Freemantle in INQUIRY: The Journal of Health Care Organization, Provision, and Financing
