# Supplementary material for: Preferences for Advanced Therapy Medicinal Products: Understanding the Published Literature on the Value of Innovative Health Interventions
Source: Inquiry. 2025 Nov 1;62:00469580251390763. doi: 10.1177/00469580251390763 (PMC12580532; doi:10.1177/00469580251390763)
Supplement: sj-docx-1-inq-10.1177_00469580251390763 – Supplemental material for Preferences for Advanced Therapy Medicinal Products: Understanding the Published Literature on the Value of Innovative Health Interventions [file sj-docx-1-inq-10.1177_00469580251390763.docx]

## Appendix 1

**Search terms for genomic therapies and cell/tissue-based therapies.**

**Genomic therapies.**

**OVID Database**

Embase 1974 to 2021 May 28 and 1^st^ January 2021 to 3^rd^ March 2024

| 1 | Exp personalised medicine/ or exp genetic therapy/ |
| --- | --- |
| 2 | ("gen* therap*" or genetic or genomic* or "personali#ed adj2 medicine" or "precision adj2 medicine" or "stratified adj2 medicine" or inherited).tw |
| 3 | 1 and 2 |
| 4 | (preference* or perspectiv* or economic or valu* or utilit* or attribute* or demand* or "cost-effective*" or "cost effective*" or "cost-benefit" or "cost benefit" or "cost-utility" or "cost utility" or "willingness to pay" or "willingness-to-pay" or "WTP" or "BWS" or "best worst scaling" or "VAS" or "visual analogue scale" or "swing weighting" or "time trade off" or "time trade-off" or "TTO" or "standard gamble" or "SG" or "discrete choice experiment" or "DCE" or "conjoint analysis" or "CA" or "contingent valuation" or "CV" or "threshold technique" or "person trade-off" or "person trade off" or "PTO" or "vignette").tw. |
| 5 | 3 and 4 |
| 6 | exp animals/ or exp invertebrate/ or animal experiment/ or animal model/ |
| 7 | 5 not 6 |

MedLine – Ovid Medline ® ALL- 1946 to May 28 2021 and 1^st^ January 2021 to 3^rd^ March 2024

| 1 | exp precision medicine/ or exp genetic testing/ or exp genetic therapy/ |
| --- | --- |
| 2 | ("gen* therap*" or genetic or genomic* or "personali#ed adj2 medicine" or "precision adj2 medicine" or "stratified adj2 medicine" or inherited).tw |
| 3 | 1 and 2 |
| 4 | (preference* or perspectiv* or economic or valu* or utilit* or attribute* or demand* or "cost-effective*" or "cost effective*" or "cost-benefit" or "cost benefit" or "cost-utility" or "cost utility" or "willingness to pay" or "willingness-to-pay" or "WTP" or "BWS" or "best worst scaling" or "VAS" or "visual analogue scale" or "swing weighting" or "time trade off" or "time trade-off" or "TTO" or "standard gamble" or "SG" or "discrete choice experiment" or "DCE" or "conjoint analysis" or "CA" or "contingent valuation" or "CV" or "threshold technique" or "person trade-off" or "person trade off" or "PTO" or "vignette").tw |
| 5 | 3 and 4 |
| 6 | exp animals/ or exp invertebrate/ or animal experiment/ or animal model/ |
| 7 | 5 not 6 |

AMED (Allied and Complementary Medicine) 1985 to May 2021 and 1^st^ January 2021 to 3^rd^ March 2024

| 1 | exp genes/ or exp allied health/ or exp genetics/ or exp evidence based medicine/ or exp health policy/ |
| --- | --- |
| 2 | ("gen* therap*" or genetic or genomic* or "personali#ed adj2 medicine" or "precision adj2 medicine" or "stratified adj2 medicine" or inherited).tw |
| 3 | 1 and 2 |
| 4 | (preference* or perspectiv* or economic or valu* or utilit* or attribute* or demand* or "cost-effective*" or "cost effective*" or "cost-benefit" or "cost benefit" or "cost-utility" or "cost utility" or "willingness to pay" or "willingness-to-pay" or "WTP" or "BWS" or "best worst scaling" or "VAS" or "visual analogue scale" or "swing weighting" or "time trade off" or "time trade-off" or "TTO" or "standard gamble" or "SG" or "discrete choice experiment" or "DCE" or "conjoint analysis" or "CA" or "contingent valuation" or "CV" or "threshold technique" or "person trade-off" or "person trade off" or "PTO" or "vignette").tw |
| 5 | 3 and 4 |
| 6 | exp animals/ or exp invertebrate/ or animal experiment/ or animal model/ |
| 7 | 5 not 6 |

Global Health 1973 to 2021 Week 21 and 1^st^ January 2021 to 3^rd^ March 2024

| 1 | Exp gene therapy/ |
| --- | --- |
| 2 | ("gen* therap*" or genetic or genomic* or "personali#ed adj2 medicine" or "precision adj2 medicine" or "stratified adj2 medicine" or inherited).tw |
| 3 | 1 and 2 |
| 4 | (preference* or perspectiv* or economic or valu* or utilit* or attribute* or demand* or "cost-effective*" or "cost effective*" or "cost-benefit" or "cost benefit" or "cost-utility" or "cost utility" or "willingness to pay" or "willingness-to-pay" or "WTP" or "BWS" or "best worst scaling" or "VAS" or "visual analogue scale" or "swing weighting" or "time trade off" or "time trade-off" or "TTO" or "standard gamble" or "SG" or "discrete choice experiment" or "DCE" or "conjoint analysis" or "CA" or "contingent valuation" or "CV" or "threshold technique" or "person trade-off" or "person trade off" or "PTO" or "vignette").tw |
| 5 | 3 and 4 |
| 6 | exp animals/ or exp invertebrate/ or animal experiment/ or animal model/ |
| 7 | 5 not 6 |

**ProQuest**

ProQuest Central-1970-Current

| 1 | MJMESH.EXACT("Genetic Therapy") OR MJMESH.EXACT("Genetic Testing") OR MJMESH.EXACT("Genetic Engineering") OR MJMESH.EXACT("Precision Medicine") |
| --- | --- |
| AND | ("gen* therap*" or genetic or genomic* or "personali#ed NEAR2 medicine" or "precision NEAR2 medicine" or "stratified NEAR2 medicine" or inherited).NOFT |
| AND | (preference* or perspectiv* or economic or valu* or utilit* or attribute* or demand* or "cost-effective*" or "cost effective*" or "cost-benefit" or "cost benefit" or "cost-utility" or "cost utility" or "willingness to pay" or "willingness-to-pay" or "WTP" or "BWS" or "best worst scaling" or "VAS" or "visual analogue scale" or "swing weighting" or "time trade off" or "time trade-off" or "TTO" or "standard gamble" or "SG" or "discrete choice experiment" or "DCE" or "conjoint analysis" or "CA" or "contingent valuation" or "CV" or "threshold technique" or "person trade-off" or "person trade off" or "PTO" or "vignette").NOFT  Filter: humans |
| Overall | (MJMESH.EXACT("Genetic Therapy") OR MJMESH.EXACT("Genetic Testing") OR MJMESH.EXACT("Genetic Engineering") OR MJMESH.EXACT("Precision Medicine")) AND noft(("gen* therap*" or genetic or genomic* or "personali#ed NEAR2 medicine" or "precision NEAR2 medicine" or "stratified NEAR2 medicine" or inherited)) AND noft((preference* or perspectiv* or economic or valu* or utilit* or attribute* or demand* or "cost-effective*" or ("cost effective" OR "cost effectively" OR "cost effectiveness") or "cost-benefit" or "cost benefit" or "cost-utility" or "cost utility" or "willingness to pay" or "willingness-to-pay" or "WTP" or "BWS" or "best worst scaling" or "VAS" or "visual analogue scale" or "swing weighting" or "time trade off" or "time trade-off" or "TTO" or "standard gamble" or "SG" or "discrete choice experiment" or "DCE" or "conjoint analysis" or "CA" or "contingent valuation" or "CV" or "threshold technique" or "person trade-off" or "person trade off" or "PTO" or "vignette")) |

Social Sciences Premium Collection (1914-Current)

| 1 | MAINSUBJECT.EXACT("Gene therapy") OR MAINSUBJECT.EXACT("Genetic testing") OR MAINSUBJECT.EXACT("Precision medicine") |
| --- | --- |
| 2 | ("gen* therap*" or genetic or genomic* or "personali#ed NEAR2 medicine" or "precision NEAR2 medicine" or "stratified NEAR2 medicine" or inherited).NOFT |
| 4 | (preference* or perspectiv* or economic or valu* or utilit* or attribute* or demand* or "cost-effective*" or "cost effective*" or "cost-benefit" or "cost benefit" or "cost-utility" or "cost utility" or "willingness to pay" or "willingness-to-pay" or "WTP" or "BWS" or "best worst scaling" or "VAS" or "visual analogue scale" or "swing weighting" or "time trade off" or "time trade-off" or "TTO" or "standard gamble" or "SG" or "discrete choice experiment" or "DCE" or "conjoint analysis" or "CA" or "contingent valuation" or "CV" or "threshold technique" or "person trade-off" or "person trade off" or "PTO" or "vignette").NOFT |
| Overall | (MAINSUBJECT.EXACT("Gene therapy") OR MAINSUBJECT.EXACT("Genetic testing") OR MAINSUBJECT.EXACT("Precision medicine")) AND noft(("gen* therap*" or genetic or genomic* or "personali#ed NEAR2 medicine" or "precision NEAR2 medicine" or "stratified NEAR2 medicine" or inherited)) AND noft((preference* or perspectiv* or economic or valu* or utilit* or attribute* or demand* or "cost-effective*" or "cost effective*" or "cost-benefit" or "cost benefit" or "cost-utility" or "cost utility" or "willingness to pay" or "willingness-to-pay" or "WTP" or "BWS" or "best worst scaling" or "VAS" or "visual analogue scale" or "swing weighting" or "time trade off" or "time trade-off" or "TTO" or "standard gamble" or "SG" or "discrete choice experiment" or "DCE" or "conjoint analysis" or "CA" or "contingent valuation" or "CV" or "threshold technique" or "person trade-off" or "person trade off" or "PTO" or "vignette")) |

Applied Social Sciences Index and Abstracts (ASSIA) via ProQuest – to current

| 1 | MAINSUBJECT.EXACT.EXPLODE("Genetic engineering") OR MAINSUBJECT.EXACT.EXPLODE("Gene therapy") OR MAINSUBJECT.EXACT.EXPLODE("Clinical decision making") |
| --- | --- |
| 2 | ("gen* therap*" or genetic or genomic* or "personali#ed NEAR2 medicine" or "precision NEAR2 medicine" or "stratified NEAR2 medicine" or inherited).NOFT |
| 3 | (preference* or perspectiv* or economic or valu* or utilit* or attribute* or demand* or "cost-effective*" or "cost effective*" or "cost-benefit" or "cost benefit" or "cost-utility" or "cost utility" or "willingness to pay" or "willingness-to-pay" or "WTP" or "BWS" or "best worst scaling" or "VAS" or "visual analogue scale" or "swing weighting" or "time trade off" or "time trade-off" or "TTO" or "standard gamble" or "SG" or "discrete choice experiment" or "DCE" or "conjoint analysis" or "CA" or "contingent valuation" or "CV" or "threshold technique" or "person trade-off" or "person trade off" or "PTO" or "vignette").NOFT |
| Overall | (MAINSUBJECT.EXACT.EXPLODE("Genetic engineering") OR MAINSUBJECT.EXACT.EXPLODE("Gene therapy") OR MAINSUBJECT.EXACT.EXPLODE("Clinical decision making")) AND noft(("gen* therap*" OR genetic OR genomic* OR "personali#ed NEAR2 medicine" OR "precision NEAR2 medicine" OR "stratified NEAR2 medicine" OR inherited)) AND noft((preference* or perspectiv* or economic or valu* or utilit* or attribute* or demand* or "cost-effective*" or "cost effective*" or "cost-benefit" or "cost benefit" or "cost-utility" or "cost utility" or "willingness to pay" or "willingness-to-pay" or "WTP" or "BWS" or "best worst scaling" or "VAS" or "visual analogue scale" or "swing weighting" or "time trade off" or "time trade-off" or "TTO" or "standard gamble" or "SG" or "discrete choice experiment" or "DCE" or "conjoint analysis" or "CA" or "contingent valuation" or "CV" or "threshold technique" or "person trade-off" or "person trade off" or "PTO" or "vignette")) |

**EBSCO Host**

EconLit – to current

| 1 | SU Health or Public Health |
| --- | --- |
| AND | ("gen* therap*" or genetic or genomic* or "personali?ed N2 medicine" or "precision N2 medicine" or "stratified N2 medicine" or inherited).tx |
| AND | (preference* or perspectiv* or economic or valu* or utilit* or attribute* or demand* or "cost-effective*" or "cost effective*" or "cost-benefit" or "cost benefit" or "cost-utility" or "cost utility" or "willingness to pay" or "willingness-to-pay" or "WTP" or "BWS" or "best worst scaling" or "VAS" or "visual analogue scale" or "swing weighting" or "time trade off" or "time trade-off" or "TTO" or "standard gamble" or "SG" or "discrete choice experiment" or "DCE" or "conjoint analysis" or "CA" or "contingent valuation" or "CV" or "threshold technique" or "person trade-off" or "person trade off" or "PTO" or "vignette").tx |
| **Overall** | ( SU Health or Public Health ) AND TX ( ("gen* therap*" or genetic or genomic* or "personali?ed N2 medicine" or "precision N2 medicine" or "stratified N2 medicine" or inherited) ) AND TX ( (preference* or perspectiv* or economic or valu* or utilit* or attribute* or demand* or "cost-effective*" or "cost effective*" or "cost-benefit" or "cost benefit" or "cost-utility" or "cost utility" or "willingness to pay" or "willingness-to-pay" or "WTP" or "BWS" or "best worst scaling" or "VAS" or "visual analogue scale" or "swing weighting" or "time trade off" or "time trade-off" or "TTO" or "standard gamble" or "SG" or "discrete choice experiment" or "DCE" or "conjoint analysis" or "CA" or "contingent valuation" or "CV" or "threshold technique" or "person trade-off" or "person trade off" or "PTO" or "vignette") ) |

Web of Science Core Collection – to current

| 1 | **TI=**("gen* therap*" or "personali?ed NEAR2 medicine" or "precision NEAR2 medicine" or "stratified NEAR2 medicine" or inherited) |
| --- | --- |
| AND | **TI=**(preference* or perspectiv* or economic or valu* or utilit* or attribute* or demand* or "cost-effective*" or "cost effective*" or "cost-benefit" or "cost benefit" or "cost-utility" or "cost utility" or "willingness to pay" or "willingness-to-pay" or "WTP" or "BWS" or "best worst scaling" or "VAS" or "visual analogue scale" or "swing weighting" or "time trade off" or "time trade-off" or "TTO" or "standard gamble" or "SG" or "discrete choice experiment" or "DCE" or "conjoint analysis" or "CA" or "contingent valuation" or "CV" or "threshold technique" or "person trade-off" or "person trade off" or "PTO" or "vignette") |
|  | #2 AND #1  Filter: economics |

SCOPUS – to current

| 1 | ("gen* therap*" or genetic or genomic* or "personali?ed W2 medicine" or "precision W2 medicine" or "stratified W2 medicine" or inherited).ti,ab,kw |
| --- | --- |
| AND | (preference* or perspectiv* or economic or valu* or utilit* or attribute* or demand* or "cost-effective*" or "cost effective*" or "cost-benefit" or "cost benefit" or "cost-utility" or "cost utility" or "willingness to pay" or "willingness-to-pay" or "WTP" or "BWS" or "best worst scaling" or "VAS" or "visual analogue scale" or "swing weighting" or "time trade off" or "time trade-off" or "TTO" or "standard gamble" or "SG" or "discrete choice experiment" or "DCE" or "conjoint analysis" or "CA" or "contingent valuation" or "CV" or "threshold technique" or "person trade-off" or "person trade off" or "PTO" or "vignette").ti,ab,kw  Filter: economics |

**Cell and tissue-based therapies search strategies.**

The following searches were run for cell and tissue-based therapies:

**OVID Database**

Embase 1974 to 2021 May 28 and 1^st^ January 2021 to 3^rd^ March 2024

| 1 | exp "Cell- and Tissue-Based Therapy"/ or exp Cell Differentiation/ or exp Stem Cells/ or exp Regenerative Medicine/ or exp Tissue Engineering/ or exp Embryo, Mammalian/ or exp Tissue Transplantation/ or exp Mesenchymal Stem Cells/ or exp Tissue Engineering/ or exp Cells, Cultured/ or exp Biomedical Engineering/ or exp Stromal Cells/ or exp Induced Pluripotent Stem Cells/ or exp Regeneration/ or exp Human Embryonic Stem Cells/ or exp Pluripotent Stem Cells/ or exp Fetal Stem Cells/ or exp Embryonic Stem Cells/ or Cells/ or exp Hematopoietic Stem Cells/ or exp Adult Stem Cells/ or exp Totipotent Stem Cells/ or exp Multipotent Stem Cells/ or exp Allogeneic Cells/ or exp Transplantation, Autologous/ or exp Bioengineering/ or exp Cell Transplantation/ or exp Stromal Cells/ or exp Stem Cell Research/ |
| --- | --- |
| 2 | (preference* or perspectiv* or economic or valu* or utilit* or attribute* or demand* or "cost-effective*" or "cost effective*" or "cost-benefit" or "cost benefit" or "cost-utility" or "cost utility" or "willingness to pay" or "willingness-to-pay" or "WTP" or "BWS" or "best worst scaling" or "VAS" or "visual analogue scale" or "swing weighting" or "time trade off" or "time trade-off" or "TTO" or "standard gamble" or "SG" or "discrete choice experiment" or "DCE" or "conjoint analysis" or "CA" or "contingent valuation" or "CV" or "threshold technique" or "person trade-off" or "person trade off" or "PTO" or "vignette").tw |
| 3 | 1 and 2 |
| 4 | exp animals/ or exp invertebrate/ or animal experiment/ or animal model/ |
| 5 | 3 not 4 |
| 6 | limit 5 to "economics (maximizes sensitivity)" |

MedLine -Ovid Medline® ALL 1946 to May 28 2021 and 1^st^ January 2021 to 3^rd^ March 2024

| 1 | exp "Cell- and Tissue-Based Therapy"/ or exp Cell Differentiation/ or exp Stem Cells/ or exp Regenerative Medicine/ or exp Tissue Engineering/ or exp Embryo, Mammalian/ or exp Tissue Transplantation/ or exp Mesenchymal Stem Cells/ or exp Tissue Engineering/ or exp Cells, Cultured/ or exp Biomedical Engineering/ or exp Stromal Cells/ or exp Induced Pluripotent Stem Cells/ or exp Regeneration/ or exp Human Embryonic Stem Cells/ or exp Pluripotent Stem Cells/ or exp Fetal Stem Cells/ or exp Embryonic Stem Cells/ or Cells/ or exp Hematopoietic Stem Cells/ or exp Adult Stem Cells/ or exp Totipotent Stem Cells/ or exp Multipotent Stem Cells/ or exp Allogeneic Cells/ or exp Transplantation, Autologous/ or exp Bioengineering/ or exp Cell Transplantation/ or exp Stromal Cells/ or exp Stem Cell Research/ |
| --- | --- |
| 2 | (preference* or perspectiv* or economic or valu* or utilit* or attribute* or demand* or "cost-effective*" or "cost effective*" or "cost-benefit" or "cost benefit" or "cost-utility" or "cost utility" or "willingness to pay" or "willingness-to-pay" or "WTP" or "BWS" or "best worst scaling" or "VAS" or "visual analogue scale" or "swing weighting" or "time trade off" or "time trade-off" or "TTO" or "standard gamble" or "SG" or "discrete choice experiment" or "DCE" or "conjoint analysis" or "CA" or "contingent valuation" or "CV" or "threshold technique" or "person trade-off" or "person trade off" or "PTO" or "vignette").tw |
| 3 | 1 and 2 |
| 4 | exp animals/ or exp invertebrate/ or animal experiment/ or animal model/ |
| 5 | 3 not 4 |
| 6 | limit 5 to "economics (maximizes sensitivity)" |

AMED (Allied and Complementary Medicine) 1985 to May 2021- Original SLR

| 1 | exp "Cell- and Tissue-Based Therapy"/ or exp Cell Differentiation/ or exp Stem Cells/ or exp Regenerative Medicine/ or exp Tissue Engineering/ or exp Embryo, Mammalian/ or exp Tissue Transplantation/ or exp Mesenchymal Stem Cells/ or exp Tissue Engineering/ or exp Cells, Cultured/ or exp Biomedical Engineering/ or exp Stromal Cells/ or exp Induced Pluripotent Stem Cells/ or exp Regeneration/ or exp Human Embryonic Stem Cells/ or exp Pluripotent Stem Cells/ or exp Fetal Stem Cells/ or exp Embryonic Stem Cells/ or Cells/ or exp Hematopoietic Stem Cells/ or exp Adult Stem Cells/ or exp Totipotent Stem Cells/ or exp Multipotent Stem Cells/ or exp Allogeneic Cells/ or exp Transplantation, Autologous/ or exp Bioengineering/ or exp Cell Transplantation/ or exp Stromal Cells/ or exp Stem Cell Research/ |
| --- | --- |
| 2 | (preference* or perspectiv* or economic or valu* or utilit* or attribute* or demand* or "cost-effective*" or "cost effective*" or "cost-benefit" or "cost benefit" or "cost-utility" or "cost utility" or "willingness to pay" or "willingness-to-pay" or "WTP" or "BWS" or "best worst scaling" or "VAS" or "visual analogue scale" or "swing weighting" or "time trade off" or "time trade-off" or "TTO" or "standard gamble" or "SG" or "discrete choice experiment" or "DCE" or "conjoint analysis" or "CA" or "contingent valuation" or "CV" or "threshold technique" or "person trade-off" or "person trade off" or "PTO" or "vignette").tw |
| 3 | 1 and 2 |
| 4 | exp animals/ or exp invertebrate/ or animal experiment/ or animal model/ |
| 5 | 3 not 4 |

AMED (Allied and Complementary Medicine) January 2021 to March 2024 – Updated SLR

| 1 | exp "Cell therapy" |
| --- | --- |
| 2 | (preference* or perspectiv* or economic or valu* or utilit* or attribute* or demand* or "cost-effective*" or "cost effective*" or "cost-benefit" or "cost benefit" or "cost-utility" or "cost utility" or "willingness to pay" or "willingness-to-pay" or "WTP" or "BWS" or "best worst scaling" or "VAS" or "visual analogue scale" or "swing weighting" or "time trade off" or "time trade-off" or "TTO" or "standard gamble" or "SG" or "discrete choice experiment" or "DCE" or "conjoint analysis" or "CA" or "contingent valuation" or "CV" or "threshold technique" or "person trade-off" or "person trade off" or "PTO" or "vignette").tw |
| 3 | 1 and 2 |
| 4 | exp animals/ or exp invertebrate/ or animal experiment/ or animal model/ |
| 5 | 3 not 4 |

Global health 1973 to 2024 week 9

| 1 | Cell- and Tissue-Based Therapy or Cell Differentiation or Stem Cells or Regenerative Medicine or Tissue Engineering or Tissue Transplantation or Allogeneic Cells or Autologous cells.mp |
| --- | --- |
| 2 | (preference* or perspectiv* or economic or valu* or utilit* or attribute* or demand* or "cost-effective*" or "cost effective*" or "cost-benefit" or "cost benefit" or "cost-utility" or "cost utility" or "willingness to pay" or "willingness-to-pay" or "WTP" or "BWS" or "best worst scaling" or "VAS" or "visual analogue scale" or "swing weighting" or "time trade off" or "time trade-off" or "TTO" or "standard gamble" or "SG" or "discrete choice experiment" or "DCE" or "conjoint analysis" or "CA" or "contingent valuation" or "CV" or "threshold technique" or "person trade-off" or "person trade off" or "PTO" or "vignette").tw |
| 3 | 1 and 2 |
| 4 | exp animals/ or exp invertebrate/ or animal experiment/ or animal model/ |
| 5 | 3 not 4 |

**ProQuest**

ProQuest Central- 1970-Current

| 1 | MJMESH.EXACT("Induced Pluripotent Stem Cells") OR MJMESH.EXACT("Adult Stem Cells") OR MJMESH.EXACT("Embryonic Stem Cells") OR MJMESH.EXACT("Tissue Transplantation") OR MJMESH.EXACT("Hematopoietic Stem Cells") OR MJMESH.EXACT("Mesenchymal Stem Cells") OR MJMESH.EXACT("Regenerative Medicine") OR MJMESH.EXACT("Bioengineering") OR MJMESH.EXACT("Hematopoietic Stem Cell Transplantation") OR MJMESH.EXACT("Mesenchymal Stem Cell Transplantation") OR MJMESH.EXACT("Allogeneic Cells") OR MJMESH.EXACT("Adult Germline Stem Cells") OR MJMESH.EXACT("Multipotent Stem Cells") OR MJMESH.EXACT("Investigative Techniques") OR MJMESH.EXACT("Cell Engineering") OR MJMESH.EXACT("Stem Cell Research") OR MJMESH.EXACT("Pluripotent Stem Cells") OR MJMESH.EXACT("Stem Cell Transplantation") OR MJMESH.EXACT("Cord Blood Stem Cell Transplantation") OR MJMESH.EXACT("Transplantation, Autologous") OR MJMESH.EXACT("Hematopoietic Stem Cell Mobilization") OR MJMESH.EXACT("Therapeutics") OR MJMESH.EXACT("Stem Cells") OR MJMESH.EXACT("Tissue Engineering") OR MJMESH.EXACT("Human Embryonic Stem Cells") OR MJMESH.EXACT("Stromal Cells") OR MJMESH.EXACT("Fetal Stem Cells") |
| --- | --- |
| AND | (preference* or perspectiv* or economic or valu* or utilit* or attribute* or demand* or "cost-effective*" or "cost effective*" or "cost-benefit" or "cost benefit" or "cost-utility" or "cost utility" or "willingness to pay" or "willingness-to-pay" or "WTP" or "BWS" or "best worst scaling" or "VAS" or "visual analogue scale" or "swing weighting" or "time trade off" or "time trade-off" or "TTO" or "standard gamble" or "SG" or "discrete choice experiment" or "DCE" or "conjoint analysis" or "CA" or "contingent valuation" or "CV" or "threshold technique" or "person trade-off" or "person trade off" or "PTO" or "vignette").NOFT  Filter: humans |
| Overall | (MJMESH.EXACT("Induced Pluripotent Stem Cells") OR MJMESH.EXACT("Adult Stem Cells") OR MJMESH.EXACT("Embryonic Stem Cells") OR MJMESH.EXACT("Tissue Transplantation") OR MJMESH.EXACT("Hematopoietic Stem Cells") OR MJMESH.EXACT("Mesenchymal Stem Cells") OR MJMESH.EXACT("Regenerative Medicine") OR MJMESH.EXACT("Bioengineering") OR MJMESH.EXACT("Hematopoietic Stem Cell Transplantation") OR MJMESH.EXACT("Mesenchymal Stem Cell Transplantation") OR MJMESH.EXACT("Allogeneic Cells") OR MJMESH.EXACT("Adult Germline Stem Cells") OR MJMESH.EXACT("Multipotent Stem Cells") OR MJMESH.EXACT("Investigative Techniques") OR MJMESH.EXACT("Cell Engineering") OR MJMESH.EXACT("Stem Cell Research") OR MJMESH.EXACT("Pluripotent Stem Cells") OR MJMESH.EXACT("Stem Cell Transplantation") OR MJMESH.EXACT("Cord Blood Stem Cell Transplantation") OR MJMESH.EXACT("Transplantation, Autologous") OR MJMESH.EXACT("Hematopoietic Stem Cell Mobilization") OR MJMESH.EXACT("Therapeutics") OR MJMESH.EXACT("Stem Cells") OR MJMESH.EXACT("Tissue Engineering") OR MJMESH.EXACT("Human Embryonic Stem Cells") OR MJMESH.EXACT("Stromal Cells") OR MJMESH.EXACT("Fetal Stem Cells")) AND noft((preference* OR perspectiv* OR economic OR valu* OR utilit* OR attribute* OR demand* OR "cost-effective*" OR "cost effective*" OR "cost-benefit" OR "cost benefit" OR "cost-utility" OR "cost utility" OR "willingness to pay" OR "willingness-to-pay" OR "WTP" OR "BWS" OR "best worst scaling" OR "VAS" OR "visual analogue scale" OR "swing weighting" OR "time trade off" OR "time trade-off" OR "TTO" OR "standard gamble" OR "SG" OR "discrete choice experiment" OR "DCE" OR "conjoint analysis" OR "CA" OR "contingent valuation" OR "CV" OR "threshold technique" OR "person trade-off" OR "person trade off" OR "PTO" or "vignette") ) |

Social Sciences Premium Collection (1914-Current)

| 1 | MAINSUBJECT.EXACT("Stem cells") OR MAINSUBJECT.EXACT("Tissue engineering") |
| --- | --- |
| AND | (preference* or perspectiv* or economic or valu* or utilit* or attribute* or demand* or "cost-effective*" or "cost effective*" or "cost-benefit" or "cost benefit" or "cost-utility" or "cost utility" or "willingness to pay" or "willingness-to-pay" or "WTP" or "BWS" or "best worst scaling" or "VAS" or "visual analogue scale" or "swing weighting" or "time trade off" or "time trade-off" or "TTO" or "standard gamble" or "SG" or "discrete choice experiment" or "DCE" or "conjoint analysis" or "CA" or "contingent valuation" or "CV" or "threshold technique" or "person trade-off" or "person trade off" or "PTO" or "vignette").NOFT |
| Overall | (MAINSUBJECT.EXACT("Stem cells") OR MAINSUBJECT.EXACT("Tissue engineering")) AND noft((preference* OR perspectiv* OR economic OR valu* OR utilit* OR attribute* OR demand* OR "cost-effective*" OR "cost effective*" OR "cost-benefit" OR "cost benefit" OR "cost-utility" OR "cost utility" OR "willingness to pay" OR "willingness-to-pay" OR "WTP" OR "BWS" OR "best worst scaling" OR "VAS" OR "visual analogue scale" OR "swing weighting" OR "time trade off" OR "time trade-off" OR "TTO" OR "standard gamble" OR "SG" OR "discrete choice experiment" OR "DCE" OR "conjoint analysis" OR "CA" OR "contingent valuation" OR "CV" OR "threshold technique" OR "person trade-off" OR "person trade off" OR "PTO" or "vignette")) |

Applied Social Sciences Index and Abstracts (ASSIA) via ProQuest – to current – Original search

| 1 | MAINSUBJECT.EXACT.EXPLODE("Peripheral stem cells") OR MAINSUBJECT.EXACT.EXPLODE("Haematopoietic stem cells") OR MAINSUBJECT.EXACT.EXPLODE("Stem cells") |
| --- | --- |
| AND | (preference* or perspectiv* or economic or valu* or utilit* or attribute* or demand* or "cost-effective*" or "cost effective*" or "cost-benefit" or "cost benefit" or "cost-utility" or "cost utility" or "willingness to pay" or "willingness-to-pay" or "WTP" or "BWS" or "best worst scaling" or "VAS" or "visual analogue scale" or "swing weighting" or "time trade off" or "time trade-off" or "TTO" or "standard gamble" or "SG" or "discrete choice experiment" or "DCE" or "conjoint analysis" or "CA" or "contingent valuation" or "CV" or "threshold technique" or "person trade-off" or "person trade off" or "PTO" or "vignette").NOFT |
| Overall | (MAINSUBJECT.EXACT.EXPLODE("Peripheral stem cells") OR MAINSUBJECT.EXACT.EXPLODE("Haematopoietic stem cells") OR MAINSUBJECT.EXACT.EXPLODE("Stem cells")) AND noft((preference* OR perspectiv* OR economic OR valu* OR utilit* OR attribute* OR demand* OR "cost-effective*" OR "cost effective*" OR "cost-benefit" OR "cost benefit" OR "cost-utility" OR "cost utility" OR "willingness to pay" OR "willingness-to-pay" OR "WTP" OR "BWS" OR "best worst scaling" OR "VAS" OR "visual analogue scale" OR "swing weighting" OR "time trade off" OR "time trade-off" OR "TTO" OR "standard gamble" OR "SG" OR "discrete choice experiment" OR "DCE" OR "conjoint analysis" OR "CA" OR "contingent valuation" OR "CV" OR "threshold technique" OR "person trade-off" OR "person trade off" OR "PTO" or "vignette")) |

Applied Social Sciences Index and Abstracts (ASSIA) via ProQuest – to current - Updated SLR (March 2024)

| 1 | MAINSUBJECT.EXACT.EXPLODE("Biology") OR MAINSUBJECT.EXACT.EXPLODE("Stem cells") |
| --- | --- |
| AND | (preference* or perspectiv* or economic or valu* or utilit* or attribute* or demand* or "cost-effective*" or "cost effective*" or "cost-benefit" or "cost benefit" or "cost-utility" or "cost utility" or "willingness to pay" or "willingness-to-pay" or "WTP" or "BWS" or "best worst scaling" or "VAS" or "visual analogue scale" or "swing weighting" or "time trade off" or "time trade-off" or "TTO" or "standard gamble" or "SG" or "discrete choice experiment" or "DCE" or "conjoint analysis" or "CA" or "contingent valuation" or "CV" or "threshold technique" or "person trade-off" or "person trade off" or "PTO" or "vignette").NOFT |
| Overall | (MAINSUBJECT.EXACT.EXPLODE("Peripheral stem cells") OR MAINSUBJECT.EXACT.EXPLODE("Haematopoietic stem cells") OR MAINSUBJECT.EXACT.EXPLODE("Stem cells")) AND noft((preference* OR perspectiv* OR economic OR valu* OR utilit* OR attribute* OR demand* OR "cost-effective*" OR "cost effective*" OR "cost-benefit" OR "cost benefit" OR "cost-utility" OR "cost utility" OR "willingness to pay" OR "willingness-to-pay" OR "WTP" OR "BWS" OR "best worst scaling" OR "VAS" OR "visual analogue scale" OR "swing weighting" OR "time trade off" OR "time trade-off" OR "TTO" OR "standard gamble" OR "SG" OR "discrete choice experiment" OR "DCE" OR "conjoint analysis" OR "CA" OR "contingent valuation" OR "CV" OR "threshold technique" OR "person trade-off" OR "person trade off" OR "PTO" or "vignette")) |

**EBSCO Host**

EconLit – to current

| 1 | "advanced therapy medicinal product" or "advanced therapy" or "cell-based therapy" or "cellular therapy" or "cell therapy" or "stem cell therapy" or "stem cell transplant" or "biomaterials" or "bioengineering" or "bioengineered" or "regenerative medicine" or "regenerative engineering" or "biotherapeutics" or "cell engineering" or "tissue repair" or "tissue product" or "cell transplantation" or "tissue transplantation" or "tissue engineering" |
| --- | --- |
| AND | (preference* or perspectiv* or economic or valu* or utilit* or attribute* or demand* or "cost-effective*" or "cost effective*" or "cost-benefit" or "cost benefit" or "cost-utility" or "cost utility" or "willingness to pay" or "willingness-to-pay" or "WTP" or "BWS" or "best worst scaling" or "VAS" or "visual analogue scale" or "swing weighting" or "time trade off" or "time trade-off" or "TTO" or "standard gamble" or "SG" or "discrete choice experiment" or "DCE" or "conjoint analysis" or "CA" or "contingent valuation" or "CV" or "threshold technique" or "person trade-off" or "person trade off" or "PTO" or "vignette").tx |
| **Overall** | ( "advanced therapy medicinal product" or "advanced therapy" or "cell-based therapy" or "cellular therapy" or "cell therapy" or "stem cell therapy" or "stem cell transplant" or "biomaterials" or "bioengineering" or "bioengineered" or "regenerative medicine" or "regenerative engineering" or "biotherapeutics" or "cell engineering" or "tissue repair" or "tissue product" or "cell transplantation" or "tissue transplantation" or "tissue engineering" ) AND TX ( (preference* or perspectiv* or economic or valu* or utilit* or attribute* or demand* or "cost-effective*" or "cost effective*" or "cost-benefit" or "cost benefit" or "cost-utility" or "cost utility" or "willingness to pay" or "willingness-to-pay" or "WTP" or "BWS" or "best worst scaling" or "VAS" or "visual analogue scale" or "swing weighting" or "time trade off" or "time trade-off" or "TTO" or "standard gamble" or "SG" or "discrete choice experiment" or "DCE" or "conjoint analysis" or "CA" or "contingent valuation" or "CV" or "threshold technique" or "person trade-off" or "person trade off" or "PTO" or "vignette") ) |

Web of Science Core Collection – to current

| 1 | TI= "advanced therapy medicinal product" or "advanced therapy" or "cell-based therapy" or "cellular therapy" or "cell therapy" or "stem cell therapy" or "stem cell transplant" or "biomaterials" or "bioengineering" or "bioengineered" or "regenerative medicine" or "regenerative engineering" or "biotherapeutics" or "cell engineering" or "tissue repair" or "tissue product" or "cell transplantation" or "tissue transplantation" or "tissue engineering" |
| --- | --- |
| AND | **TI=**(preference* or perspectiv* or economic or valu* or utilit* or attribute* or demand* or "cost-effective*" or "cost effective*" or "cost-benefit" or "cost benefit" or "cost-utility" or "cost utility" or "willingness to pay" or "willingness-to-pay" or "WTP" or "BWS" or "best worst scaling" or "VAS" or "visual analogue scale" or "swing weighting" or "time trade off" or "time trade-off" or "TTO" or "standard gamble" or "SG" or "discrete choice experiment" or "DCE" or "conjoint analysis" or "CA" or "contingent valuation" or "CV" or "threshold technique" or "person trade-off" or "person trade off" or "PTO" or "vignette")  Filter: economics |
| Overall | #2 AND #1 |

SCOPUS – to current

| 1 | "advanced therapy medicinal product" or "advanced therapy" or "cell-based therapy" or "cellular therapy" or "cell therapy" or "stem cell therapy" or "stem cell transplant" or "biomaterials" or "bioengineering" or "bioengineered" or "regenerative medicine" or "regenerative engineering" or "biotherapeutics" or "cell engineering" or "tissue repair" or "tissue product" or "cell transplantation" or "tissue transplantation" or "tissue engineering". ti,ab,kw |
| --- | --- |
| AND | (preference* or perspectiv* or economic or valu* or utilit* or attribute* or demand* or "cost-effective*" or "cost effective*" or "cost-benefit" or "cost benefit" or "cost-utility" or "cost utility" or "willingness to pay" or "willingness-to-pay" or "WTP" or "BWS" or "best worst scaling" or "VAS" or "visual analogue scale" or "swing weighting" or "time trade off" or "time trade-off" or "TTO" or "standard gamble" or "SG" or "discrete choice experiment" or "DCE" or "conjoint analysis" or "CA" or "contingent valuation" or "CV" or "threshold technique" or "person trade-off" or "person trade off" or "PTO" or "vignette").ti,ab,kw  Filter: Economics |
