# Supplementary material for: Preferences for Advanced Therapy Medicinal Products: Understanding the Published Literature on the Value of Innovative Health Interventions
Source: Inquiry. 2025 Nov 1;62:00469580251390763. doi: 10.1177/00469580251390763 (PMC12580532; doi:10.1177/00469580251390763)
Supplement: sj-docx-2-inq-10.1177_00469580251390763 – Supplemental material for Preferences for Advanced Therapy Medicinal Products: Understanding the Published Literature on the Value of Innovative Health Interventions [file sj-docx-2-inq-10.1177_00469580251390763.docx]

## Appendix 2

### Compliance checklist

The following table presents the compliance checklist of the identified discrete choice experiments.

Table 5. Compliance checklist of identified discrete choice experiments

| **Checklist Components** | **Witkop et al., 2021** | **Sepulveda et al., 2023** | **Sun et al., 2020** | **Boeri et al., 2021** | **Birch et al., 2022** | **Liu et al., 2023** |
| --- | --- | --- | --- | --- | --- | --- |
| 1. Was a well-defined research question stated and is conjoint analysis an appropriate method for answering it? | | | | | | |
| Well defined research question and testable hypothesis | Yes | Yes | Yes | Yes | Yes | Yes |
| Study perspective described and placement of study | Yes | Yes | Yes | Yes | Yes | Yes |
| Rationale for conjoint analysis | Yes – to measure relative importance of each attribute | Yes – to select preferred treatment alternatives | Yes – to assess preferences for different haemophilia A treatments | Yes – explore physician preferences and quantify trade-offs | Yes – to quantify patient preferences for second line treatments in diffuse large B cell lymphoma | Yes – to understand how patients value benefits and risks associated with cell therapy |
| 1. Was the choice of attributes and levels supported by evidence? | | | | | | |
| Attribute identification supported by evidence | Yes – Targeted literature review and semi-structured interviews | Yes – in person focus group. Detail provided in appendix | Yes – targeted literature review and telephone interviews | Yes – chosen based on best available information | Yes – based on characteristics of emerging second line treatments | Not described |
| Attribute selection justified and consistent with theory | Yes – through an expert reference group | Yes – in person focus group. Detail provided in appendix | Yes – targeted literature review and telephone interviews | Yes – chosen based on best available information | Yes – input from clinical experts sought | Not described |
| Justification of level selection and consistent with perspective and hypothesis | No – justification of level choices not given | Yes – in person focus groups. Detail provided in appendix | Yes – targeted literature review and telephone interviews | Yes – chosen based on best available information | Yes – calibrated using clinically relevant ranges extracted from published trials | Not described |
| 1. Was the construction of tasks appropriate? | | | | | | |
| Justification of number of attributes | Partly – inferred based on input and feedback from contributors | None given | None given | Yes | Yes | None given |
| Justification of number of profiles | None given | Yes – 4 or 6 were used | No justification but states 14 choice cards | Yes – 24 choice questions | Yes – 18 questions | None given |
| Inclusion of opt-out (if relevant) | Not included – arguably not needed | Yes | Not described | Not included- arguably not needed | Not included- arguably not needed | None given |
| 1. Was the choice of experimental design justified and evaluated? | | | | | | |
| Justification of experimental design | None provided | Yes – given in appendix | None provided | Yes | Yes | None given |
| Evaluation of properties of experimental design | None provided | Yes – given in appendix | None provided | Not described | Not described | None provided |
| Appropriate number of conjoint tasks | None provided | Yes | Yes – 14 choice cards | Yes – 24 choice questions | Yes – 18 questions | Yes – 9 choice tasks |
| 1. Were preferences elicited appropriately, given the research question? | | | | | | |
| Sufficient motivation and explanation of conjoint tasks | Not entirely – a pilot was run to ascertain comprehension | Yes | Not described | Yes | Yes | Not described |
| Appropriate elicitation format including allowing for indifference | Not described | Yes | Not described | Yes | Yes | Not described |
| Other qualifying questions (e.g., strength of preferences) | No | Not described | Not described | Not described | Not described | Fixed profile representing standard of care included |
| 1. Was the data collection instrument designed appropriately? | | | | | | |
| Collection of appropriate respondent information | Yes – patient and clinical characteristics captured | Yes - patient and clinical characteristics captured | Yes - patient and clinical characteristics captured | Yes- descriptive data provided | Yes- descriptive data provided | Yes - patient and clinical characteristics captured |
| Defining of attributes and levels and provision of contextual information | Not described | Yes | Not described | Yes | Yes | Not described |
| Acceptability of level of burden on data collection | Yes | Yes | Yes | Yes | Yes | Yes |
| 1. Was the data-collection plan appropriate? | | | | | | |
| Justification of sampling strategy | Yes | Yes | No | Yes | Yes | None provided |
| Justification of mode of administration | Does not state how the survey was administered | Yes – online | Yes - online | Yes - online | Yes - online | Yes - online |
| Address ethical considerations | Yes | Yes | Refers to informed consent but no ethics | Yes | Yes | Not mentioned |
| 1. Were statistical analyses and model estimations appropriate? | | | | | | |
| Examination of respondent characteristics | Yes | Yes | Yes | Yes | Yes | Yes |
| Examination of quality of responses | Quality checks were performed | Quality checks were performed | Not described | Quality checks were performed | Quality checks were performed | Not described |
| Appropriate conduct of model estimation (e.g., clustering, subgroups) | Yes | Yes | Not described | Yes | Yes | Not described |
| 1. Were the results and conclusions valid? | | | | | | |
| Reflection of testable hypothesis and accounting for statistical uncertainty | Yes | Yes | Partly – statistical uncertainty not addressed | Yes | Yes | Partly – statistical uncertainty not addressed |
| Conclusions supported by the evidence and compared with existing studies | Yes | Yes | Not discussed | Yes | Yes | Not discussed |
| Address limitations and generalisability | Yes | Yes | Not discussed | Yes | Yes | Not discussed |
| 1. Was the study presentation clear, concise, and complete? | | | | | | |
| Adequately motivated study importance and context | Yes | Yes | Not described | Yes | Yes | Not described |
| Description of data collection instruments and methods | Partly | Yes | Not described | Yes | Yes | Not described |
| Implications of study clearly stated and understandable to a wide audience | Yes | Yes | Partly | Yes | Yes | Partly |
